# Lateral femoral notch sign and posterolateral tibial plateau fractures and their associated injuries in the setting of an anterior cruciate ligament rupture

**DOI:** 10.1007/s00402-021-04105-6

**Published:** 2021-08-02

**Authors:** Alexander Korthaus, Malte Warncke, Geert Pagenstert, Matthias Krause, Karl-Heinz Frosch, Jan Philipp Kolb

**Affiliations:** 1 Department of Trauma and Orthopaedic Surgery, University Medical Center Hamburg-Eppendorf, Martinistrasse 52, 20246 Hamburg, Germany; 2 Department of Diagnostic and Interventional Radiology and Nuclear Medicine, University Medical Center Hamburg-Eppendorf, Martinistrasse 52, 20246 Hamburg, Germany; 3 CLARAHOF Clinic of Orthopaedic Surgery, University of Basel, Clarahofweg 19a, 4058 Basel, Switzerland; 4 Department of Trauma Surgery, Orthopaedics and Sports Traumatology, BG Hospital Hamburg, Bergedorfer Straße 10, 21033 Hamburg, Germany

**Keywords:** ACL rupture, Posterolateral tibial plateau fracture, Femoral notch sign, Anterior cruciate ligament

## Abstract

**Introduction:**

ACL injury is one of the most common injuries of the knee joint in sports. As accompanying osseous injuries of the ACL rupture a femoral impression the so-called lateral femoral notch sign and a posterolateral fracture of the tibial plateau are described. However, frequency, concomitant ligament injuries and when and how to treat these combined injuries are not clear. There is still a lack of understanding with which ligamentous concomitant injuries besides the anterior cruciate ligament injury these bony injuries are associated.

**Materials and methods:**

One hundred fifteen MRI scans with proven anterior cruciate ligament rupture performed at our center were retrospectively evaluated for the presence of a meniscus, collateral ligament injury, a femoral impression, or a posterolateral impression fracture. Femoral impressions were described according to their local appearance and posterolateral tibial plateau fractures were described using the classification of Menzdorf et al.

**Results:**

In 29 cases a significant impression in the lateral femoral condyle was detected. There was a significantly increased number of lateral meniscal (41.4% vs. 18.6% *p* = 0.023) and medial ligament (41.4% vs. 22.1%; *p* = 0.040) injuries in the group with a lateral femoral notch sign. 104 patients showed a posterolateral bone bruise or fracture of the tibial plateau. Seven of these required an intervention according to Menzdorf et al. In the group of anterior cruciate ligament injuries with posterolateral tibial plateau fracture significantly more lateral meniscus injuries were seen (*p* = 0.039).

**Conclusion:**

In the preoperative planning of ACL rupture accompanied with a positive femoral notch sign, attention should be paid to possible medial collateral ligament and lateral meniscus injuries. As these are more likely to occur together. A posterolateral impression fracture of the tibial plateau is associated with an increased likelihood of the presence of a lateral meniscal injury. This must be considered in surgical therapy and planning and may be the indication for necessary early surgical treatment.

## Introduction

**Fig. 1:**
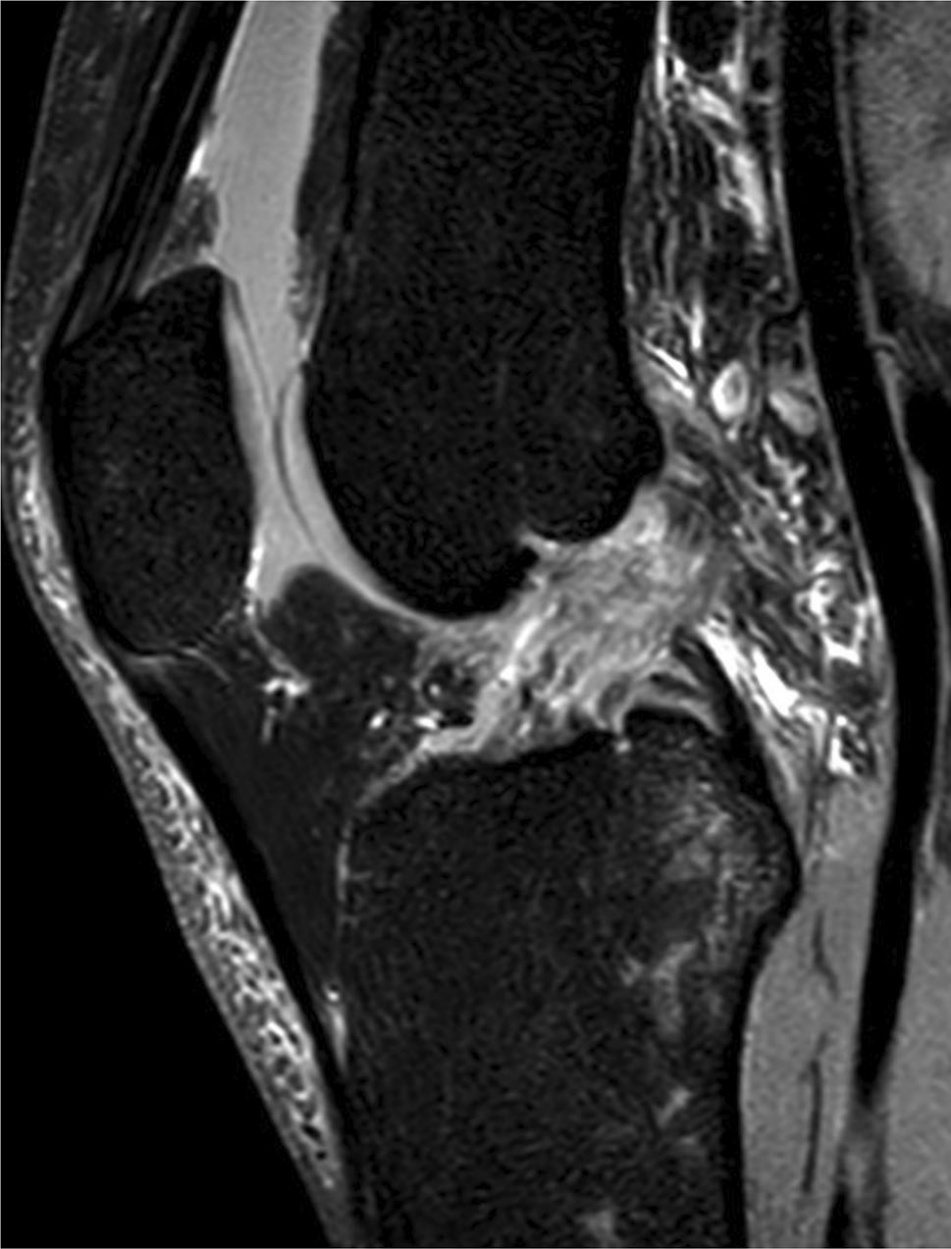
MRI scan of a left Knee in saggital plane with a ACL rupture

The anterior cruciate ligament (ACL) rupture is one of the most common sports injuries (Fig. 1) [1]. As part of the mechanism of the injury, a valgus/internal rotation mechanism results in ventral subluxation of the tibia, and the lateral femoral condyle may strike the posterolateral tibial plateau. Depending on the force applied, there may be anything from edema to fractures in these areas [2–5]. Furthermore, this mechanism of injury is also associated with injury to the lateral meniscus posterior horn [6].

**Fig. 2:**
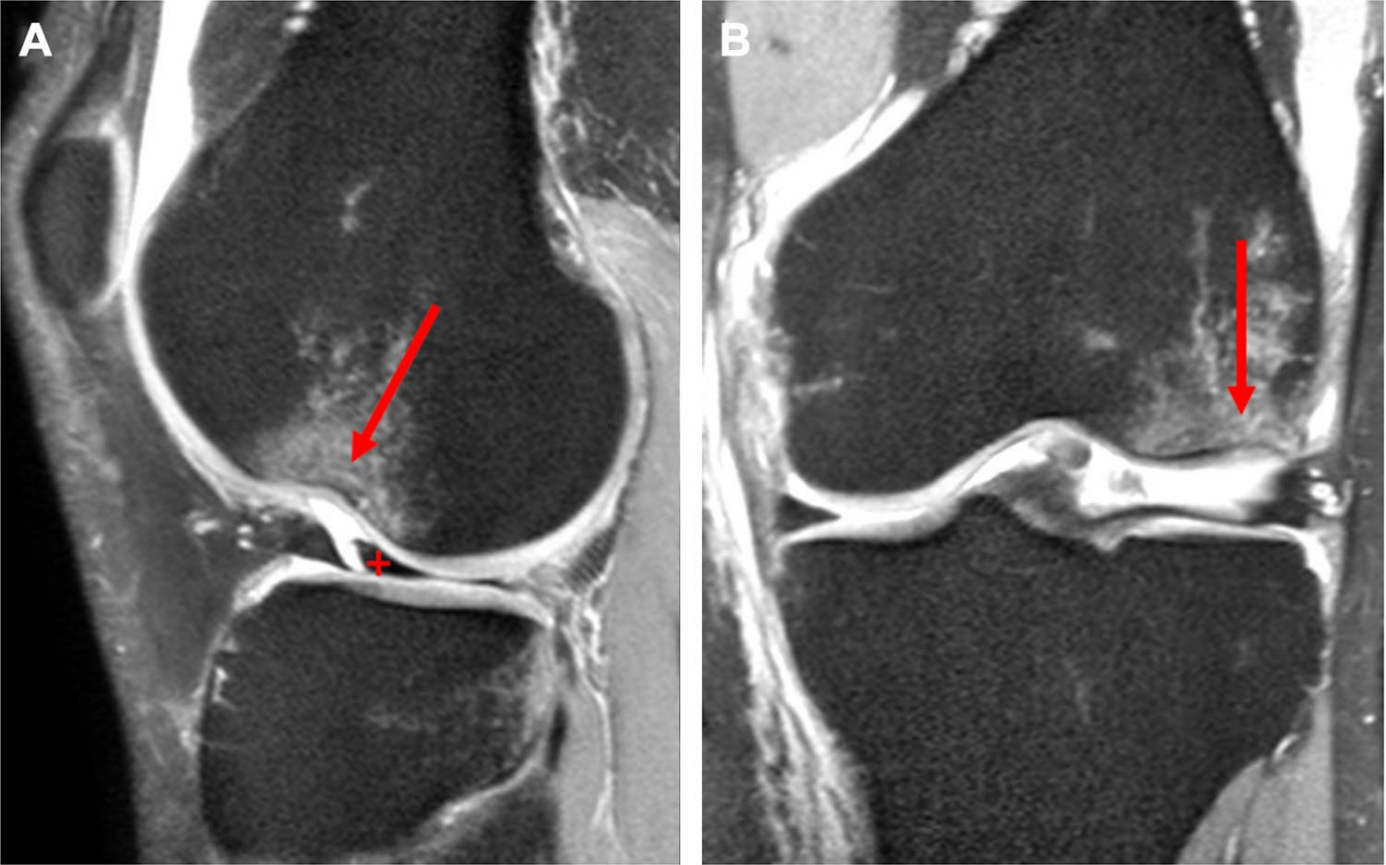
Illustration of an MRI of a left knee joint in **A** saggital section plane showing a lateral notch sign (arrow) and a basket handle tear (cross) of the external meniscus and **B** in coronal section plane showing a lateral notch sign

**Fig. 3:**
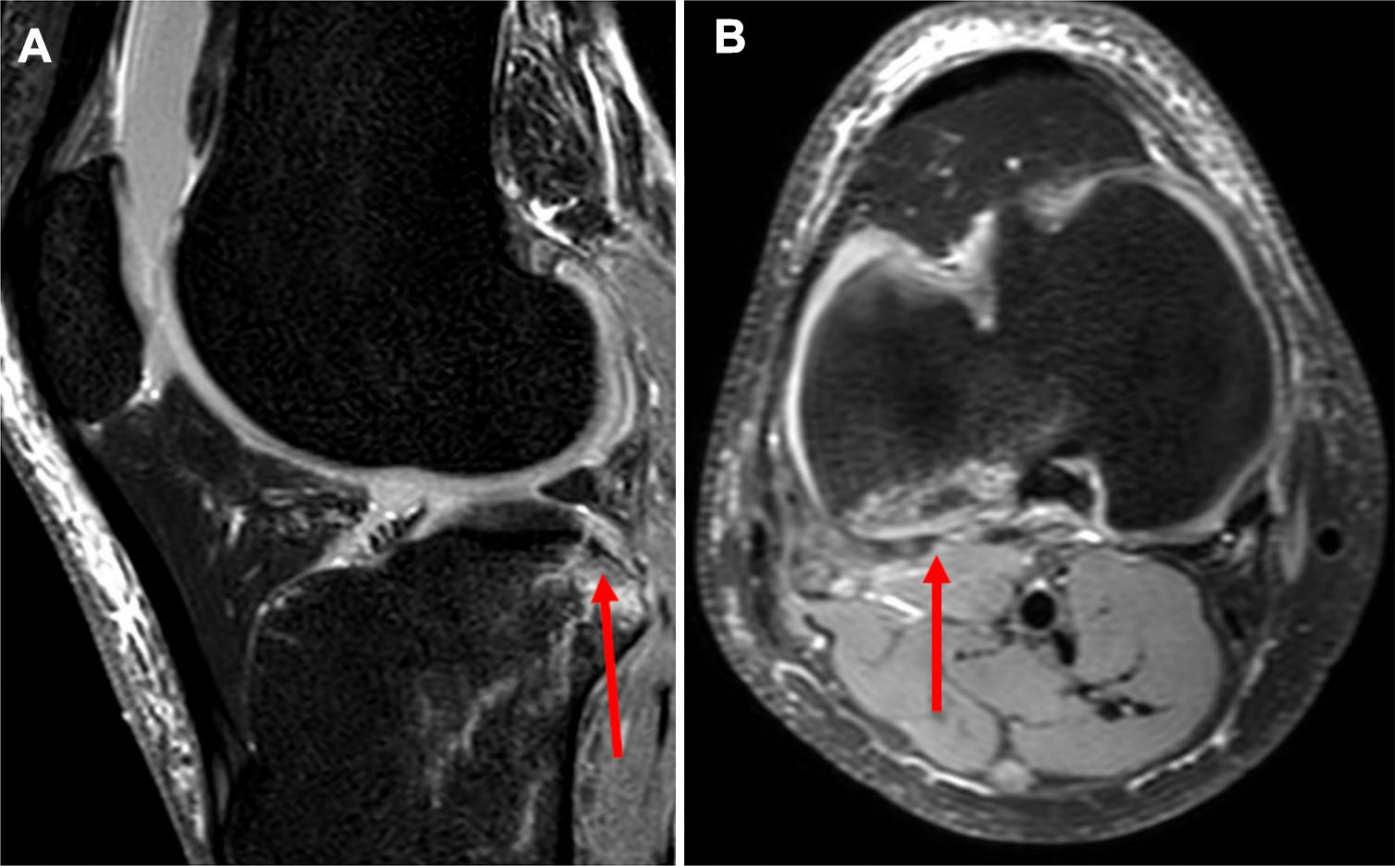
Illustration of an MRI of a left knee joint in **A** saggital section plane showing a posterolateral fracture of the tibial plateau (arrow) and **B** transversal section plane showing a posterolateral fracture of the tibial plasteau

In at least 3 out of 4 cases, in addition to the ACL rupture, MRI can demonstrate bone edema in the posterolateral tibial plateau region and at the lateral femoral condyle [7]. Described in the literature is the so-called lateral femoral notch sign (LFNS) with an impression of the lateral distal femur as well as the posterolateral fracture of the tibial plateau, which is typically localized in the posterolateral-lateral (PLL) and posterolateral-central (PLC) segments according to the 10 segment classification of the German Knee Society [2, 8] (Figs. 2, 3).

Both fractures are considered predictive of ACL injury [2, 9–11]. For the LFNS, an incidence of 20–60% has been reported depending on the literature [10–14].

Due to the deformity of the joint surface in weight-bearing areas, early-onset osteoarthrosis may occur in the long term [2, 12, 15]. Therefore, accompanying injuries in particular must also be recognized and, if necessary, treated.

To our knowledge, no valid data exist yet for the incidence of posterolateral fractures requiring intervention in this group. In our opinion, there is still a lack of understanding with which ligamentous concomitant injuries besides the anterior cruciate ligament injury this osseous injury is associated. Furthermore, it is completely unclear when a posterolateral tibial fracture and when a fracture occurs in the lateral femoral articular surface.

We therefore reviewed 115 MRI scans with proven anterior cruciate ligament rupture for LFNS and the posterolateral fracture of the tibial plateau and their associated injuries.

## Methods

A total of 115 MRI scans using a 3-Tesla MRI (Ingenia 3T Phillips) with confirmed anterior cruciate ligament rupture between July 2016 and October 2020 were included (Fig. 1).

MRI scans were followed up for the presence of meniscal injury, collateral ligament injury, significant impression (> 2 mm) of the lateral femoral condyle, the LFNS (Fig. 2), and posterolateral fracture of the tibial plateau (Fig. 3).

No differences were made regarding the morphology of the collateral ligament injury (e.g., proximal/ distal avulsion, intraligamentous rupture) or the meniscal injuries (e.g., flap tear, root tear etc.).

**Fig. 4:**
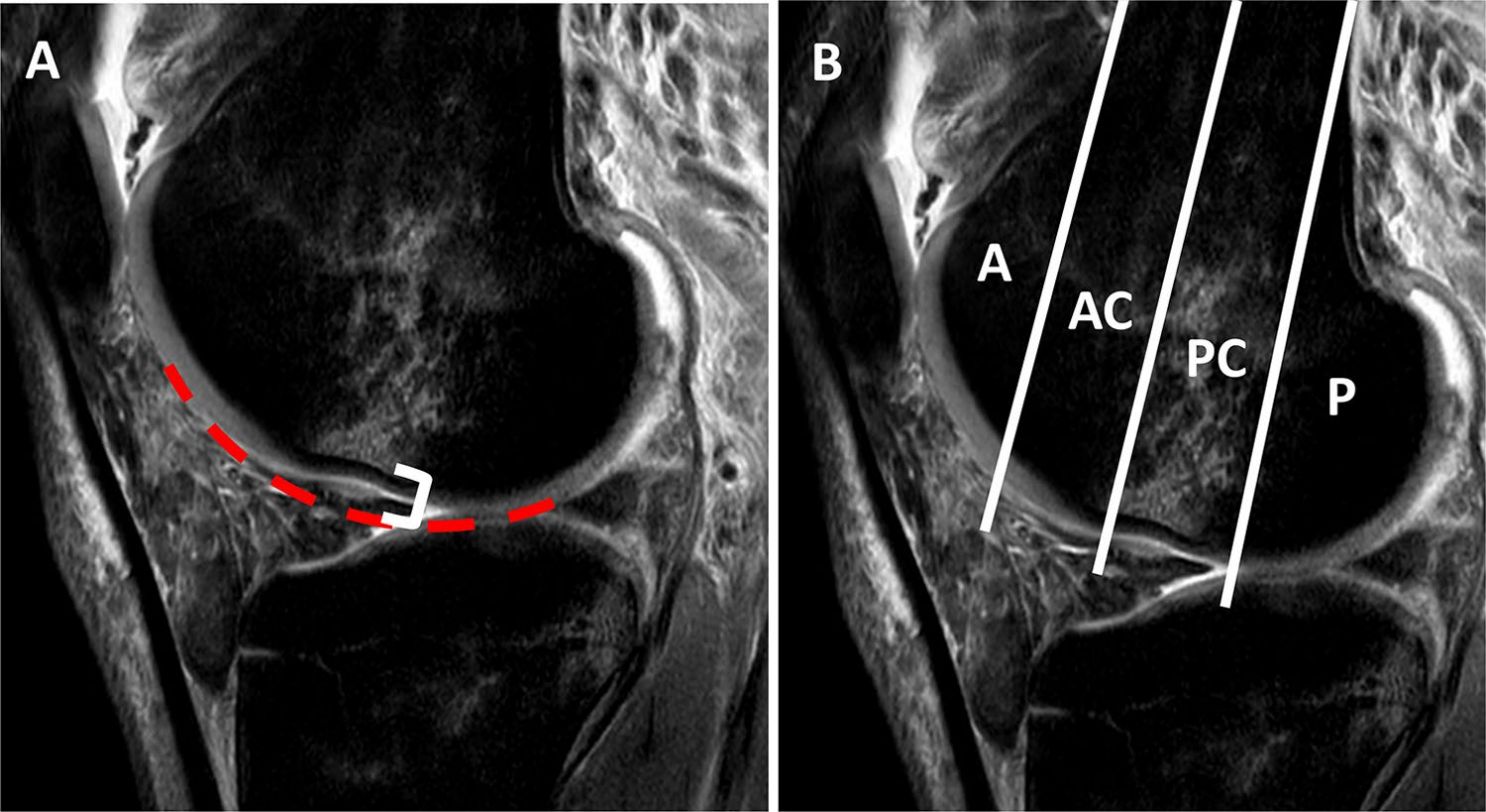
**A** Measurement of the lateral femoral impression: the red elliptical line shows the femoral articular surface without the impression, the white bracket represents the impression depth starting from the red line to the bottom of the osseous impression, measured at the deepest point. **B** Determination of the localization of the femoral impression. The sagittal femoral condyle is divided into 4 quadrants by drawing 3 lines at the deepest point of the impression. The most ventral and dorsal lines represented the distal extension of the femoral shaft. The median bisects the central region. *A* anterior quadrant, *AC* antero-central quadrant, *PC* postero-central quadrant, *P* posterior quadrant

The femoral impression was characterized on the basis of the depth of the impression in the sagittal planes. A line corresponding to the shape of the femoral condyle without impression was drawn and the depth of the impression was measured from this line to the osseous base at the deepest point (Fig. 4). To describe the location of the impression the sagittal femoral condyle was divided into 4 quadrants by drawing 3 lines at the lowest point of the impression. The most ventral and dorsal lines represented the distal extension of the femoral shaft. The median bisects the central region (Fig. 4).

**Fig. 5:**
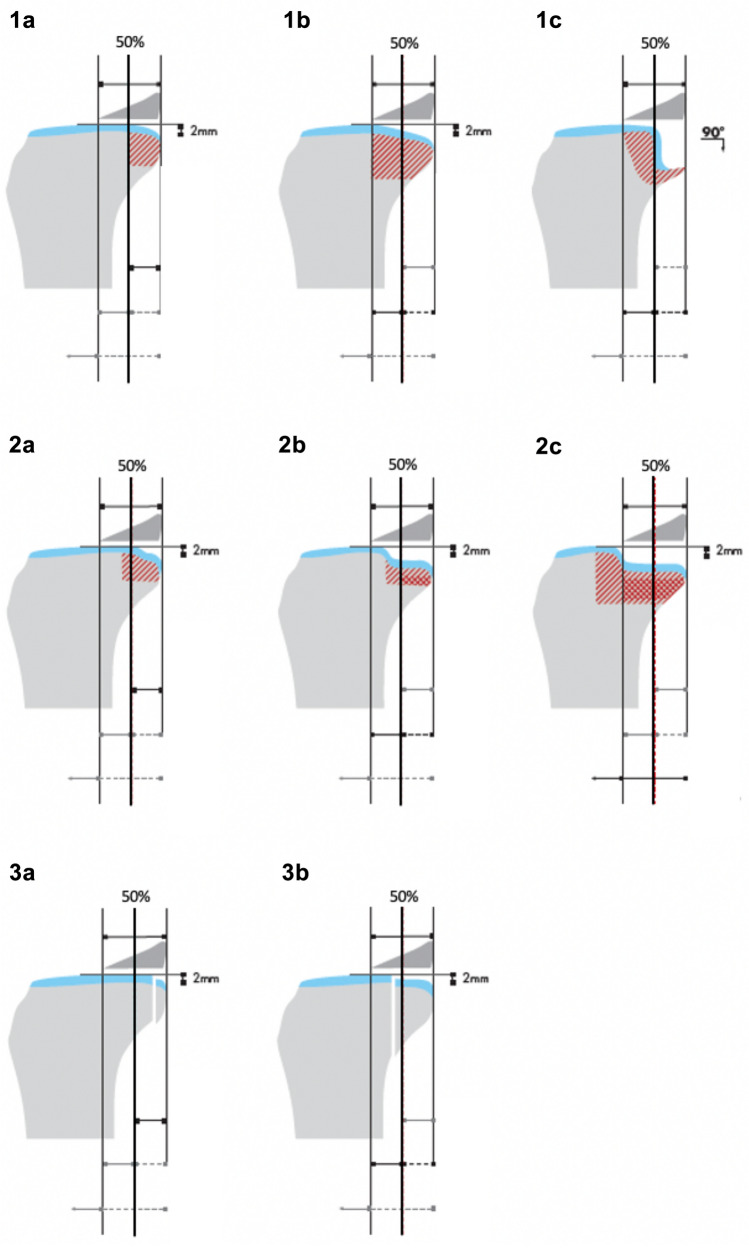
Classification of posterolateral impression fractures modified of Menzdorf et al. [2]. Three different entities are distinguished: rim fractures (1), depression fractures (2) and shear fractures (3). These 3 groups are each subdivided. The decisive factor is whether more than 50% of the posterior horn of the lateral meniscus is supported by bone and whether there is a dislocation of less than 2 mm depth. If this is the case, the respective fracture type is assigned the letter **a**. If this is not the case, the fracture form is assigned the letter **b**, whereby the criteria mentioned decide on the need for surgery. Within the group of “rim fractures” there is another separate entity: the “90° deformity”, to which the letter **c** is assigned. Within the group of depression fractures, there is a subgroup that is also assigned **a**, **c**. In this group, 100% of the posterior horn of the lateral meniscus are not supported by the posterolateral plateau and there is a dislocation of more than 2 mm depth

The classification of posterolateral impression fractures was based on the classification of Menzdorf et al. (Fig. 5) [2]. Here, basically 3 different entities are distinguished: rim fractures (1), depression fractures (2) and shear fractures (3). These 3 groups are each subdivided into subgroups. The decisive factor is whether more than 50% of the posterior horn of the lateral meniscus is supported by bone and whether there is a dislocation of less than 2 mm depth. If this is the case, the respective fracture type is assigned the letter a. If this is not the case, the fracture form is assigned the letter b, whereby the criteria mentioned decide on the need for surgery. Within the group of “rim fractures” there is another separate entity: the “90° deformity”, to which the letter c is assigned. Within the group of depression fractures, there is a subgroup that is also assigned a, c. In this group, 100% of the posterior horn of the lateral meniscus are not supported by the posterolateral plateau and there is a dislocation of more than 2 mm depth.

The study was conducted after a positive ethical vote of the resident ethics board.

### Statistical analysis

A Shapiro–Wilk normality test and Kolmogorov–Smirnov test were performed to determine if the data were normally distributed. Pearson and Spearman coefficient was used for correlation of parametric and nonparametric data, respectively. Comparison of binominal data was performed using Fisher’s exact test. Comparison of two paired groups with parametric and nonparametric data was performed using paired *t* test and Wilcoxon signed rank test, respectively. Comparison of two unpaired groups with parametric and nonparametric data was performed using independent *t* test and Mann–Whitney *U* test, respectively. The level of significance for all tests was set at *p* ≤ 0.05. All data were analyzed using IBM SPSS Statistics version 26.0 (IBM, Armonk, NY).

## Results

In the entire patient collective, 104 patients (90.43%) showed posterolateral bone bruise with minor impaction of the tibial head and 29 (25.3%) significant impressions in the lateral femoral condyle.

Seven posterolateral fractures of the tibial plateau required intervention according to Menzdorf et al. [2]. This results in an incidence of 6.1% for posterolateral fractures requiring intervention in the setting of anterior cruciate ligament injuries in our collective.

Overall, we saw 29 impressions (25.3%) in the lateral femoral condyle. Within this group, significantly more males (*n* = 23) than females (*n* = 6) were affected (*p* = 0.016). Furthermore, the patient population with femoral impression tended to be older (33.3 ± 11.7) compared to the group without significant impression (30.6 ± 9.2; *p* = 0.03).

Analysis of concomitant injury in patients with (*n* = 29) versus without (*n* = 86) femoral impression showed that within the group with femoral impression there were significantly more lateral meniscal injuries (41.4% vs. 18.6%; *p* = 0.023) as well as medial ligament injuries (41.4% vs. 22.1%; *p* = 0.040) (Table 1).

**Table 1:** Demographics and concomitant injuries in patients with (*N* = 29) vs. without (*N* = 86) bump in 115 patients with ruptured anterior crucial ligament

Parameter	Patients with posterolateral impaction	Patients without posterolateral impaction	P value
Gender			
Male	23	46	0.016
Female	6	40	
Age [years]	33.3 ± 11.7	30.6 ± 9.2	0.003^‡^
Medial meniscus tear			
Yes	6	21	0.803^†^
No	23	65	
Lateral meniscus tear			
Yes	12	16	0.023^†^
No	17	70	
MCL rupture			
Yes	12	19	0.040^†^
No	17	67	
LCL rupture			
Yes	5	27	0.159^†^
No	24	59	

^†^Using Fisher’s exact test

^‡^Using Mann–Whitney *U* test

Impression of the femoral condyle was localized antero-central in one patient (3.4%), antero-central and postero-central in 13 patients (44.8%), and isolated to the postero-central quadrant in 14 patients (48.3%). One patient (3.4%) showed the impression localized in 3 quadrants. No patient had an impression in the posterior quadrant.

Posterolateral fracture of the tibial head requiring intervention according to Menzdorf et al. was not seen in any patient within the group with lateral femoral impression. There were 27 rim fractures type 1a and one depression fracture type 2a according to Menzdorf et al. which did not result in any surgical consequences [2].

The group without femoral notch sign showed a total of 76 posterolateral edema including fractures (88.4%), of which 7 (8.4%) required intervention (type 1b: *n* = 4, type 1c: *n* = 3) according to Menzdorf et al. [2]. There was no statistically significant difference between the two groups (*p* = 0.286, Table 2).

**Table 2:** Incidence and classification of poserotlateral tibial plateau fractures depending on the presence of femoral notch sign (*N* = 29) or in the absence of femoral notch sign (*N* = 86)

Parameter	Patients with femoral notch sign	Patients without femoral notch sign	*p* value
PL tibia impression			
Yes	28	76	0.286†
No	1	10	
PL tibial impression classification			
1a	27	60	0.488*
1b	0	4	
1c	0	3	
2a	1	8	
2b	0	1	
2c	0	0	
3a	0	0	
3b	0	0	

^†^Using Fisher’s exact test

^‡^Using Mann–Whitney *U* test

The mean age of the patients was 29.8 ± 5.1 years in the group of patients without posterolateral fracture and 30.7 ± 10.0 years in the group with fracture without significant difference (*p* = 0.868). However, the patients with posterolateral fracture requiring intervention showed significantly older age at 36.6 ± 9.7 years than the patients without lateral tibial plateau impression fracture requiring intervention at 29.8 ± 9.5 years (*p* = 0.029). Gender distribution showed no significant difference in both groups (*p* = 0.351).

In addition, we saw significantly more lateral meniscus injuries in the group with a posterolateral fracture (*p* = 0.039) than in the group without a posterolateral fracture. We did not see a correlation with medial meniscus injuries (*p* = 1.000), lateral collateral ligament injuries (*p* = 1.000) or medial collateral ligament injuries (*p* = 0.484, Table 3).

**Table 3:** Demographics and concomitant injuries in patients with (*N* = 104) vs. without (*N* = 11) posterolateral impaction in patients with ruptured anterior crucial ligament

Parameter	Patients with posterolateral impaction	Patients without posterolateral impaction	*p* value
Gender			
Male	62	7	1.000^†^
Female	42	4	
Age [years]	30.7 ± 10.0	29.8 ± 5.1	0.868^‡^
Lateral femoral notch sign			
Yes	28	1	0.286^†^
No	76	10	
Medial meniscus tear			
Yes	23	4	0.283^†^
No	81	7	
Lateral meniscus tear			
Yes	28	0	0.039^†^
No	76	11	
MCL rupture			
Yes	27	4	0.484^†^
No	77	7	
LCL rupture			
Yes	29	3	1.000^†^
No	75	8	

^†^Using Fisher’s exact test

^‡^Using Mann–Whitney *U* test

## Discussion

We were able to show that the incidence of fractures of the posterolateral tibial plateau requiring surgery is significantly higher than expected at 8.4%. The incidence of fractures of the lateral femoral condyle (25.3%) is within the range of 20–60% described in the literature [10, 12, 13]. The vast majority of the lateral femoral impression in our patient population is in the main weight-bearing area, which is in agreement with the data of Hoffenler et al. [13].

Here, the patients with a posterolateral fracture of the tibial plateau requiring surgery are significantly older than the patients without a fracture (*p* = 0.029). In the group of patients with a lateral femoral impression, an medial collateral ligament injury (*p* = 0.040) and an injury of the lateral meniscus (*p* = 0.023) were significantly more frequent. In addition, the presence of a posterolateral tibial plateau fracture was significantly more likely to result in a lateral meniscus injury (*p* = 0.039).

The incidence of 25.3% for femoral lateral impressions in our patient population is consistent with the recent study by Bernholt et al. and Herbst et al., who found an incidence of 25.8% and 26.3% [14, 16]. However, Bernholdt et al. defined any femoral impression > 1.5 mm as a significant impression [16]. Lucidi et al. showed that the presence of a LFNS deeper than 2 mm could be used for the preoperative identification of patients with a high risk of increased rotatory instability [17]. Furthermore, Miller et al. found an ACL injury in 70% of patients when the lateral femoral notch was deeper than 1.5 mm, and in 100% of the cases when the lateral femoral notch was deeper than 2.0 mm [18]. Further studies by Delzell et al. and Herbst et al. postulate a cut off of 2 mm for the LFNS [14, 19], which is why we decided to use a 2 mm cut off. In the current literature, there are no recommendations as to when surgical reduction of the femoral impression should be performed. But it is known that due to the deformity of the joint surface in weight-bearing areas, early-onset osteoarthrosis may occur in the long term [12, 15]. Therefore a surgical consequence results at an impression > 2 mm in our department.

If there is a significant lateral impression of the femoral condyle, the presence of a possible injury of the medial collateral ligament must be checked. In this regard, our data are consistent with the study by Bernholt et al. [16]. This does not seem surprising when the mechanism of the accident is taken into account. Thus, when the LFNS occurs, a significant valgus stress seems to occur as part of the valgus/internal rotation mechanism, leading to the corresponding impression and injury of the medial collateral ligament.

In contrast to Bernholt et al., the presence of a posterolateral tibial fracture requiring intervention according to Menzdorf et al. and a combined significant impression of the lateral femoral condyle seem to be excluded in our patient collective [2, 16]. However, in all patients with a LFNS, we saw an impression in the posterolateral quadrant of the tibial plateau, which, however, did not represent a need for surgery according to the defined criteria of Menzdorf et al. [2]. This may be due to the fact that we defined impressions of the lateral femoral condyle of > 2 mm as cut off for LFNS as previously described. In addition, the criteria previously mentioned by Menzdorf et al. for surgical treatment associated with increased instability as well as an increased posttraumatic osteoarthritis rate and thus entailing a therapeutic consequence were applied [2, 20–22]. Thus, the criteria applied in this study that have an immediate therapeutic consequence appear to not intersect.

The association with the injury of the lateral meniscus also seems logical in the light of the mechanism of an ACL injury and is consistent with the current literature. However, injury to the lateral meniscus in acute trauma also entails a therapeutic consequence and must be considered [6].

The incidence of posterolateral tibial plateau fractures requiring intervention at 6.7% is significantly higher than expected. This is even higher in the group of patients with a posterolateral bone bruise at 8.4%. So Bernholt et al. observed a lateral tibial plateau depth bone loss percentage of greater than 10% in 8.6% of all patients in primary ACL tear cohort. However, it remains unclear whether Bernholt et al. infer a need for surgery from this [16]. Therefore, it seems reasonable to use a classification from which a treatment recommendation can be derived. The classification according to Menzdorf et al. gives clear criteria for surgical treatment. Namely, dislocation of the fracture of 2 mm or more and/ or extension beyond half of the posterior horn of the lateral meniscus [2, 20, 22]. The above criteria lead to increased pivot shift and thus significant posterolateral instability in concomitant ACL rupture [2, 20].

This must be considered in surgical therapy. In our hands, the vast majority of posterolateral fractures, indicated for surgery, can be arthroscopically addressed as part of cruciate ligament reconstruction [23]. In addition, in the presence of a posterolateral fracture or even just edema, a high rate of injuries to the lateral meniscus should be expected [16, 24].

### Limitations

The patient collective of 115 patients is relatively small. Different morphologies of collateral ligament injury as well as meniscus injuries were not analyzed, so that no statement can be made about the necessity of surgical treatment. Results of clinical examinations with regard to existing instabilities are not available.

## Conclusion

ACL ruptures may be associated with osseous as well as concomitant soft tissue injuries. In particular, the incidence of fractures requiring treatment is significantly higher than expected. In our patient population, older patients tend to be affected.

In case of a positive LFNS, the presence of an lateral meniscus as well as an medial collateral ligament injury should be considered in the preoperative planning. Posterolateral fracture of the tibial plateau is associated with increased likelihood of the presence of a lateral meniscus injury.

Therefore, the presence of different form of posterolateral tibial plateau fractures requiring intervention in the setting of an anterior cruciate ligament injury must be considered in the presence of other associated soft tissue injuries.

Further studies with a larger cohort appear useful for confirmation. In particular, a prospective study of lateral femoral notch fractures in terms of functional outcome would be desirable to formulate clear criteria for surgical reduction in the future.
